# Practice-Based Management Data of Consecutive Subjects Assessed for the Median Arcuate Ligament Syndrome at a Single Tertiary Institution

**DOI:** 10.3390/clinpract14050151

**Published:** 2024-09-18

**Authors:** Stephanie Zbinden, Gabor Forgo, Nils Kucher, Stefano Barco

**Affiliations:** Department of Angiology, University Hospital Zurich, 8091 Zurich, Switzerland; stephanie.zbinden@hug.ch (S.Z.); nils.kucher@usz.ch (N.K.); stefano.barco@usz.ch (S.B.)

**Keywords:** median arcuate ligament syndrome, Dunbar syndrome, celiac artery compression syndrome, vascular compression syndrome, color duplex ultrasound

## Abstract

Background: The pathophysiology of median arcuate ligament syndrome (MALS) is poorly understood. The diagnostic process remains inadequately standardized, with an absence of precise criteria to guide therapeutic management. Methods: We studied consecutive subjects referred to the Department of Angiology at the University Hospital of Zurich over the past 17 years due to suspected MALS. We focused on (1) the imaging criteria that led to diagnosis, notably the results of color duplex ultrasound and the consistency with different imaging tests; (2) the clinical consequences focusing on symptom resolution. Results: We included 33 subjects; in 8 subjects (24.2%), the diagnosis of MALS was retained. The median expiration peak systolic velocity (PSV) on ultrasound was 3.05 (Q1; 2.1–Q3; 3.3). To confirm the sonographic results, either a CT or MRI was performed on all patients, with consistent findings confirming a significant stenosis. Seven patients underwent surgery, all involving arcuate ligament release. Four procedures were laparoscopic, one was via laparotomy, and two were robot-assisted. Additionally, two patients required angioplasty with stenting as a secondary intervention. Only two (28.6%) of the seven operated patients experienced a relief of symptoms. None experienced a relief of symptoms following secondary angioplasty, despite stent patency. The prevalence of psychiatric disorders was comparable between patients with retained and rejected diagnoses, 38% and 36%, respectively. Conclusions: Our study confirmed sonography and CT/MRI consistency. However, most patients with MALS did not benefit from invasive treatment. The majority (83%) of patients without MALS were diagnosed with alternative conditions, mainly functional disorders.

## 1. Introduction

The European Society of Vascular Surgery describes median arcuate ligament syndrome (MALS), also known as Dunbar syndrome, as “the most common cause of single-vessel abdominal arterial stenosis” [1]. However, its epidemiology, pathophysiology, and diagnostic criteria remain largely unknown. As a consequence, the treatment is poorly standardized and based on poor evidence. 

The clinical manifestations may include heterogeneous abdominal symptoms related to the compression of the celiac artery by the median arcuate ligament. An MALS diagnosis usually follows the exclusion of other gastroenterological causes [2]. An asymptomatic compression of the celiac artery from the median arcuate ligament has a prevalence of 5% in the general population; this makes the diagnostic process more complicated, as the causal relationship between stenosis and symptoms can hardly be proven [3,4]. Due to very scarce data and unpredictable responses to treatment, even the existence of the syndrome has been questioned.

Duplex ultrasound, which enables functional testing during both inspiration and expiration, along with cross-sectional abdominal imaging (such as CT angiography or MR angiography), has been recommended as the preferred non-invasive imaging method [5]. Flow velocities at the origin of the celiac artery typically increase during expiration and normalize with inspiration. A post-stenotic dilation or aneurysm of the celiac artery is also common [6,7,8].

The treatment consists of a decompression of the celiac axis and restoration of the celiac blood flow with surgical techniques alongside optional interventional treatments (celiac artery revascularization) [9]. The resolution of symptoms following invasive treatment for stenosis is often considered the definitive confirmation of an MALS diagnosis. The current international guidelines do not specify diagnostic criteria or recommend specific treatment strategies (Supplementary Table S1) [1,10]. Therefore, the purpose of this study is to focus on the diagnostic process and identify key factors that could aid in diagnosing this condition. By addressing this gap, we aim to offer more precise data to enhance clinical practices and guide future research in this field.

## 2. Materials and Methods

Our study is a monocentric, single-arm, retrospective study of longitudinal data. We screened consecutive patients who were referred with suspected MALS at the Department of Angiology of the University Hospital Zurich over a 17-year period (2005–2022). The final data analysis was performed in January 2023. 

The study population consisted of adult patients with a clinically suspected diagnosis of MALS, as defined by the combination of clinical symptoms along with suggestive findings from duplex ultrasound and cross-sectional imaging. The routine assessment included an evaluation of typical clinical symptoms, such as unexplained epigastric and post-prandial abdominal pain, weight loss, nausea, and diarrhea. This was followed by a duplex ultrasound examination of the celiac artery, and a CT/MR angiography or conventional angiography. We considered suggestive duplex findings in the presence of a significant increase in the expiratory peak systolic velocity. Suggestive findings on cross-sectional imaging (CT/MR angiography/angiography) were represented by a focal narrowing of the celiac artery. 

The primary objective of this study was to assess the use of clinical and imaging criteria to screen for MALS in a practice-based clinical setting, also considering the correlation between color duplex ultrasound and other imaging tests. Moreover, we described the proportion and outcomes of patients with a final diagnosis who received specific therapy.

Data were collected from available medical charts, both outpatient and inpatient. These include demographics and general characteristics (such as age, sex, BMI, comorbidities), clinical presentation, imaging information (duplex ultrasound, CT angiography, MRI, angiography), supplementary investigations (gastroenterological visit) and data on endovascular/surgical treatments. Clinical outcomes were followed up to January 2023. Descriptive analyses of the baseline characteristics used counts and percentages for categorical data, whereas continuous data were expressed as mean (SD) or median and quartiles 1–3 (Q1–Q3). We used the mean for normally distributed data without significant outliers and the median for skewed data or when outliers are present. The IQR was reported alongside the median because both are resistant to outliers and together provide a comprehensive summary of the data’s central tendency and variability.

The study protocol was approved by the local ethical committee on 15 July 2022 (ID 2022-00917). In 2015, a general consent form for the use of personal data for research purposes was introduced at our institution. Personal data of patients diagnosed between 2005 and 2015 were exclusively included if the patient could not be reached to retrospectively collect a signed general consent form (article 34 of the Human Research Law, HFG). Since 2015, we have been collecting data from patients with available general consent. The study was conducted in accordance with the Declaration of Helsinki.

## 3. Results

A total of 33 patients referred for suspected MALS were assessed at the Department of Angiology of the University Hospital Zurich between 2005 and 2023. The demographic and baseline characteristics are summarized in Table 1; 27 (82%) were women and the mean age was 42.2 (SD 18) years. The median BMI was 20.0 (Q1–Q3: 18–24) kg/m^2^. In total, 31 patients (94%) were referred due to suggestive abdominal symptoms, particularly epigastric pain, after undergoing a gastroenterological evaluation that did not yield a definitive diagnosis. Two patients (6%) were referred because of a stenosis identified on CT/MRI, without having undergone a prior gastroenterological examination. 

The median length of duration of symptoms until visit to the vascular specialist was 36 (Q1–Q3: 18–84) months. Approximately half of them reported post-prandial pain, nausea, or vomiting, and one-third experienced weight loss. Diarrhea and exercise-induced pain were uncommon. 

A color duplex ultrasound was performed in almost all patients (97%) and a complementary diagnostic with cross-sectional abdominal imaging was performed in 81.8% (n = 27) of patients. In one patient, a diagnostic angiography was conducted following suggestive cross-sectional imaging, rather than using duplex ultrasound, to confirm the diagnosis.

Out of 33 subjects initially screened for suspected MALS, the diagnosis was excluded in 25 patients. Among them during expiration, the median peak systolic velocity (PSV) and end-diastolic velocity (EDV) on ultrasound were 1.5 (Q1–Q3: 1.0–1.8) and 0.35 (Q1–Q3: 0.3–0.6) m/s, respectively. During inspiration, the median PSV was 1.2 (Q1–Q3: 1.0–1.6) m/s, whereas EDV was 0.35 (Q1–Q3: 0.3–0.5) m/s, as shown in Table 2. Of those, 23 (92%) underwent a gastroenterological work-up: 19 (83%) patients were diagnosed with irritable bowel syndrome or functional dyspepsia, 1 with Wilkie’s syndrome, 1 patient with chronic non-steroidal anti-inflammatory drug abuse, and in 2 cases the pain was idiopathic. Among the remaining patients, one was diagnosed with Takayasu arteritis and one was lost to follow-up. In 19 patients (76%) without MALS, cross-sectional imaging was performed in addition to ultrasound. In two patients (11%), imaging showed celiac artery compression, though they did not present with the typical symptom constellation. In the remaining 17 patients, no stenosis was detected on cross-sectional imaging or ultrasound.

A total of eight (24.2%) diagnoses were retained due to the presence of suggestive findings on imaging, a typical clinical history, and the absence of a plausible alternative etiology. All patients with MALS had epigastric pain: in six (75%) cases, this was post-prandial, and in one subject, it was exercise-induced. Nausea and/or vomiting, and diarrhea were present in five (62.5%) patients; four reported weight loss. During expiration, the median PSV and EDV on ultrasound were 3.05 (Q1–Q3: 2.1–3.3) and 0.95 (Q1–Q3: 0.5–1.4) m/s, respectively. During inspiration, the median PSV was 1.65 (Q1–Q3: 1.6–2.5) m/s, whereas EDV was 0.85 (Q1–Q3: 0.6–1.4) m/s (Table 2). In all patients, either a CT or an MRI was performed, the findings of which were consistent with the results of color duplex ultrasound showing a significant stenosis of the celiac artery. The CT/MRI scan revealed no other arterial lesions. Among the eight patients with a retained diagnosis of MALS, three (38%) had a psychiatric disorder vs. nine (36%) in subjects without MALS (Table 3).

Seven out of eight patients with MALS underwent surgery, whereas one was lost to follow-up before receiving any treatment. The surgical procedure consisted of the release of the arcuate ligament in all patients: four were performed through laparoscopy, one through laparotomy and the two most recent cases were robot-assisted. During the laparotomy, a celiac gangliectomy was also performed. Two angioplasties with stenting (WallStent and Palmaz Blue Stent, respectively) were subsequently performed because of residual stenosis of the celiac artery at follow-up and lack of improvement of symptoms after the surgery. All patients had a follow-up period of at least 20 days, with a median follow-up of 180 days. In the postoperative color duplex ultrasound, the median PSV and EDV in the celiac artery were 1.8 m/s (Q1–Q3: 1.5–2.4) and 0.6 (Q1–Q3: 0.5–0.8) m/s, respectively. Only two (28.6%) of the seven operated patients showed relief of symptoms, but none showed relief of symptoms following secondary angioplasty, despite stent patency, as shown in Table 4.

**Figure 1:**
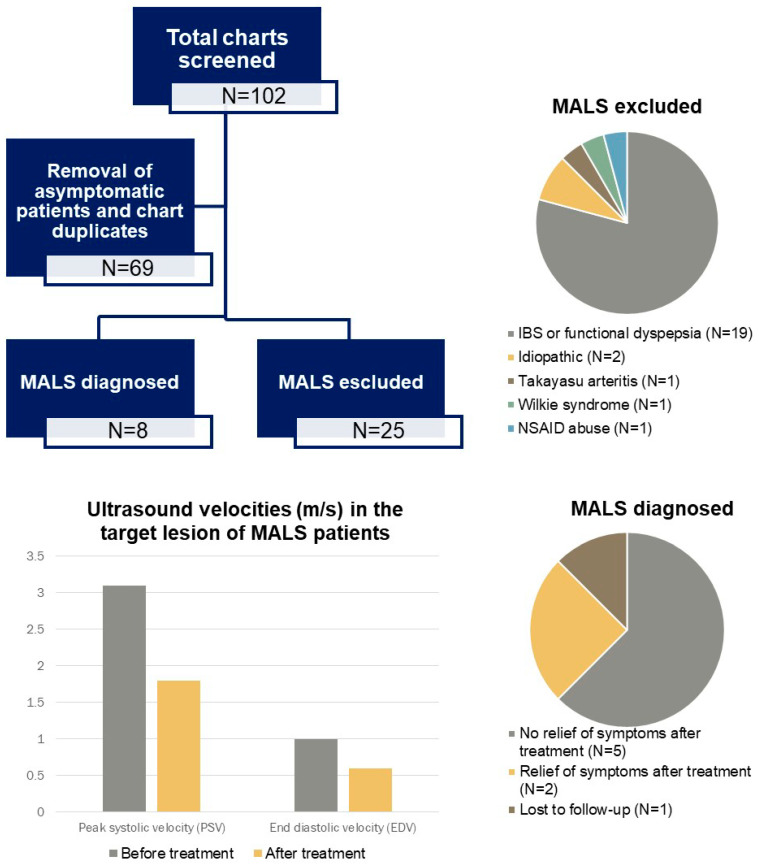
Visual abstract showing a flowchart and our findings.

**Figure 2:**
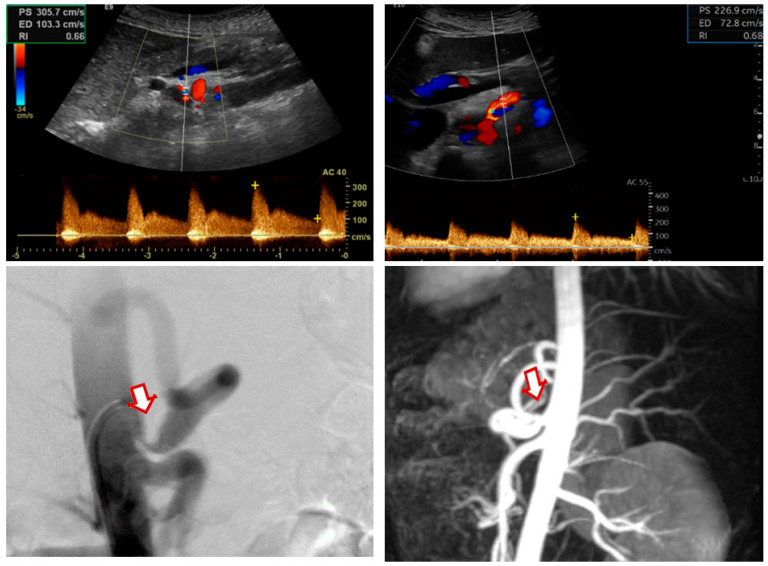
Color duplex ultrasound images showing (preoperative upper-left panel, postoperative upper-right panel) postoperative improvement in the PSV and EDV values from 305/203 cm/s to 226/72 cm/s. Digital subtraction angiography (lower-left panel) and magnetic resonance imaging (lower-right panel) showing a preoperative stenosis of the celiac artery (arrow) in a patient with MALS. PSV: peak systolic velocity; EDV: end-diastolic velocity.

**Figure 3:**
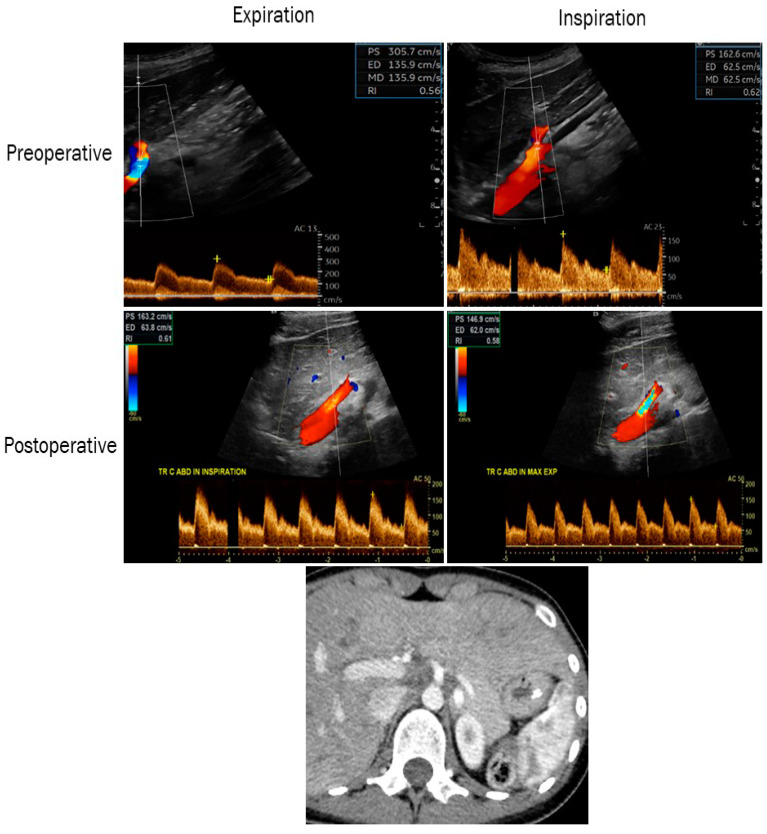
Color duplex ultrasound images showing the improvement in postoperative PSV and EDV on expiration and inspiration in a patient with MALS (preoperative upper-left 305/135 cm/s and right 162/62 cm/s, postoperative middle-left 163/63 cm/s and right 146/62 cm/s). Computed tomography (lower panel) showing a preoperative stenosis of the celiac artery in the same patient. PSV: peak systolic velocity; EDV: end-diastolic velocity.

A brief graphic summary of our results in the form of a visual abstract along with imaging samples from selected patients are shown in Figure 1, Figure 2 and Figure 3.

## 4. Discussion

We provided data from consecutive patients screened at a tertiary angiology center for median arcuate ligament syndrome (MALS) because of chronic abdominal pain. We showed that for patients in whom the diagnosis of MALS was retained, approximately one quarter of the total exhibited typical imaging findings, which were consistent across different techniques (sonography vs. CT/MRI). Furthermore, the majority of patients where MALS was excluded were diagnosed with an alternative gastroenterological condition. Unfortunately, 71% of patients with a suspected diagnosis did not benefit from the subsequent invasive treatment after the diagnostic work-up.

The diagnosis of MALS represents a challenge. The median time from symptom onset to diagnosis was 2 years, indicating a lack of disease awareness and unclear diagnostic criteria, the combination of which contributed to delayed screening. In our study, the results of color duplex ultrasound were consistent with the CT/MRI findings, both showing significant stenosis of the celiac artery without other pathologic vascular findings. The use of color duplex ultrasound in the diagnostic work-up of MALS is recommended, provided it is conducted by an experienced physician and the results are interpreted within the clinical context [10]. Our results are in line with those previously described in the literature [5,11,12]. We believe that, after color duplex ultrasound, opting for angio-MRI would be the best option in this young patient population, with diagnostic errancy and potentially multiple supplementary examinations. Indirectly, we could show that the exclusion of MALS led to further gastroenterological assessments, which ultimately lead to an alternative diagnosis. In the vast majority of cases, the alternative conditions were represented by functional disturbances (i.e., functional dyspepsia). 

Our data indicate that current treatment recommendations are being followed, which suggest a surgical approach as the first modality to release the arcuate ligament, with angioplasty considered only if symptoms persist [10]. Metz et al. performed a systematic review and found a relief of symptoms of up to 70% after this therapeutic strategy [13]. This result is significantly higher than our real-life experience (28.6%). This may suggest that some patients diagnosed with MALS actually had other underlying causes for their symptoms, given that an asymptomatic compression of the celiac artery by the arcuate ligament occurs in around 5% of the population [3,4]. Moreover, interventions, like celiac plexus block, which may improve clinical outcomes were not performed in our group of patients [14].

The interpretation of these results should take into account the relatively high prevalence of psychiatric comorbidities observed in our cohort, consistent with findings reported in the scientific literature [15]. The prevalence found is actually higher than in the general population [16]. We found no difference in prevalence between the cohort of patients rejected, and those with a confirmed diagnosis of MALS. The pattern of psychiatric illnesses encountered is mostly depression, anxiety and personality disorders, which is in line with our observation in our patient cohort. The presence of psychiatric comorbidities tends to predict a poor response to treatment [17]. In our study, the number of patients who had an intervention is too limited to make an observation. The relationship between psychiatric diseases and chronic abdominal pain appears to be confirmed. Nevertheless, their interference is still misunderstood and more studies are needed. 

This study has several limitations that warrant caution in the interpretation of our findings. The sample size was too small to perform inferential statistics. The much shorter follow-up (about 6 months) after treatment could also explain the discrepancy in our findings in comparison with the data from the literature, regarding the symptom relief after the treatment. 

## 5. Conclusions

In conclusion, our data demonstrate imaging consistency between sonography and CT/MRI findings in identifying significant celiac artery stenosis without other vascular abnormalities. Although MALS was suspected in one-third of the patients, the majority did not benefit from invasive treatment for celiac artery compression, potentially indicating an alternative diagnosis as the underlying cause of their symptoms. Given that around 5% of the general population present anatomical compression of the celiac trunk by the arcuate ligament, further research is needed to distinguish the population affected by the syndrome and define a clear algorithm. 

## Figures and Tables

**Table 1:** Baseline characteristics and clinical presentation of patients referred for suspected median arcuate ligament syndrome.

	Total Patients (n = 33)	Patients without MALS (n = 25)	Patients with MALS (n = 8)
**Demographics**			
Women, n (%)	27 (82)	21 (84)	6 (75)
Age (years), mean (SD)	42.2 (18)	44.1 (19)	36 (12)
**Comorbidities**			
BMI (kg/m^2^), median, (Q1, Q3)	20 (Q1–Q3: 18–24)	20 (Q1–Q3: 20–22)	21 (Q1–Q3: 18–25)
Psychiatric disorders, n (%)	12 (36.4)	9 (36)	3 (37.5)
Cardiovascular diseases *, n (%)	3 (9.1)	2 (8)	1 (12.5)
**Clinical presentation**			
Epigastric pain, n (%)	29 (88)	21 (84)	8 (100)
Post-prandial pain, n (%)	17 (52)	11 (44)	6 (75)
Exercise-induced pain, n (%)	3 (9.1)	2 (8)	1 (12.5)
Nausea or vomiting, n (%)	16 (48)	11 (48)	5 (62.5)
Diarrhea, n (%)	7 (21)	5 (20)	2 (25)
Weight loss, n (%	10 (30)	6 (24)	4 (50)
Duration of symptoms until visit to the vascular specialist, (months), median, (Q1, Q3)	36 (Q1–Q3: 18 –84)	33 (Q1–Q3: 12 –66)	84 (Q1–Q3: 24–240)
**Supplementary investigation**			
CT/MRI performed, n (%)	27 (82)	19 (76)	8 (100)
Gastroenterological visit, n (%)	31 (94)	23 (92)	8 (100)

* Cardiovascular diseases include coronary heart disease, cerebrovascular disease and peripheral artery disease. SD: standard deviation; BMI: body mass index; Q1–Q3: interquartile range.

**Table 2:** Ultrasound parameters.

	Patients without MALS (n = 25)	Patients with MALS (n = 8)
CA PSV (m/s) in expiratory apnea, median, (Q1, Q3)	1.5 (Q1; 1.0–Q3; 1.8)	3.05 (Q1; 2.1–Q3; 3.3)
CA PSV (m/s) in inspiratory apnea, median, (Q1, Q3)	1.2 (Q1; 1.0–Q3; 1.6)	1.65 (Q1; 1.6–Q3; 2.5)
CA EDV (m/s) in expiratory apnea, median, (Q1, Q3)	0.35 (Q1; 0.3–Q3; 0.6)	0.95 (Q1; 0.5–Q3; 1.4)
CA EDV (m/s) in inspiratory apnea, median, (Q1, Q3)	0.35 (Q1; 0.3–Q3; 0.5)	0.85 (Q1; 0.6–Q3; 1.4)
Difference PSV (m/s) expiratory–inspiratory, median	0.3	1.4
CT/MRI reports a compression, n/N (%)	2/19 (11)	8/8 (100)

MALS: median arcuate ligament syndrome; CA: celiac artery; PSV: peak systolic velocity; EDV: end-diastolic velocity; Q1–Q3, interquartile range.

**Table 3:** Details of psychiatric disorders, according to DSM-5 criteria, of patients referred for suspected median arcuate ligament syndrome and with diagnosis.

	Patients without MALS (n = 25)	Patients with MALS (n = 8)
Somatic symptom and related disorders, n (%)	7 (28)	0 (0)
Depressive disorders, n (%)	3 (12)	1 (13)
Feeding and eating disorders, n (%)	1 (4)	1 (13)
Anxiety disorders, n (%)	1 (4)	1 (13)
Personality disorders, n (%)	1 (4)	0 (0)
Obsessive compulsive disorders, n (%)	0 (0)	1 (13)
Substance-related and addictive disorders, n (%)	1 (4)	0 (0)
Any psychological or psychiatric disturbances, n (%)	9 (36)	3 (38)

DSM-5—Diagnostic and Statistical Manual of Mental Disorders, Fifth Edition.

**Table 4:** Patient follow-up after treatment.

	Patients (n = 7)
Treatment by surgery alone, n (%)	5 (71.4)
Endovascular treatment alone, n (%)	0 (0)
Endovascular and surgical treatment, n (%)	2 (28.6)
Relief of symptoms after treatment, n (%)	2 (28.6)
Time after treatment and last follow-up visit (days), median, (Q1, Q3)	180 (Q1; 27.5–Q3; 722.5)
CA PSV (m/s) after treatment, median, (Q1, Q3)	1.8 (Q1; 1.5–Q3; 2.4)
CA EDV (m/s) after treatment, median, (Q1, Q3)	0.6 (Q1; 0.5–Q3; 0.8)

CA: celiac artery; PSV: peak systolic velocity; EDV: end-diastolic velocity; Q1–Q3: interquartile range.

## Supplementary Materials

The following supporting information can be downloaded at https://www.mdpi.com/article/10.3390/clinpract14050151/s1, Table S1: Summary of existing international society recommendations on the diagnosis and management of the median arcuate ligament syndrome. References [18,19] are cited in Supplementary Materials.
