# Supplementary material for: Practice-Based Management Data of Consecutive Subjects Assessed for the Median Arcuate Ligament Syndrome at a Single Tertiary Institution
Source: Clin Pract. 2024 Sep 18;14(5):1911–20. doi: 10.3390/clinpract14050151 (PMC11417693; doi:10.3390/clinpract14050151)
Supplement: Supplementary file 1 [file clinpract-14-00151-s001.zip › clinpract-3128356-supplementary.pdf]

Supplement 1: Summary of existing international society recommendations on the diagnosis and management of the median arcuate ligament syndrome

Society	year	Diagnostic criteria	Grade	Treatment	Grade
ESVS (1)	2017	CT and exclusion	NA	NA	NA
UEG (2)	2020	US or CT scan or CE-MRA and Exclusion and Discussion in expert multidisciplinary setting	1 D	Surgical release by laparotomy or laparoscopy	2 D
SVS (3)	2021	NA	NA	Surgical release by laparotomy or laparoscopy	NA
ACR (4)	2017	CT scan or CE-MRA and angiography	NA	Surgical release by laparotomy or laparoscopy	NA

NA: Not Applicable ; ESVS : European Society of Vascular Surgery ; UEG : United European Gastroenterology ; SVS : Society for Vascular Surgery ; ACR : Appropriateness Criteria ; CT scan: Computed Tomography scan ; US : Ultrasound ; CE-MRA : Contrast-enhanced MR Angiography

1. Björck M, Koelemay M, Acosta S, Bastos Goncalves F, Kölbel T, Kolkman JJ, et al. Editor's Choice - Management of the Diseases of Mesenteric Arteries and Veins: Clinical Practice Guidelines of the European Society of Vascular Surgery (ESVS). Eur J Vasc Endovasc Surg. 2017;53(4):460-510.
2. Terlouw LG, Moelker A, Abrahamsen J, Acosta S, Bakker OJ, Baumgartner I, et al. European guidelines on chronic mesenteric ischaemia - joint United European Gastroenterology, European Association for Gastroenterology, Endoscopy and Nutrition, European Society of Gastrointestinal and Abdominal Radiology, Netherlands Association of Hepatogastroenterologists, Hellenic Society of Gastroenterology, Cardiovascular and Interventional Radiological Society of Europe, and Dutch Mesenteric Ischemia Study group clinical guidelines on the diagnosis and treatment of patients with chronic mesenteric ischaemia. United European Gastroenterol J. 2020;8(4):371-95.
3. Huber TS, Björck M, Chandra A, Clouse WD, Dalsing MC, Oderich GS, et al. Chronic mesenteric ischemia: Clinical practice guidelines from the Society for Vascular Surgery. J Vasc Surg. 2021;73(1S):87S-115S.
4. Fidelman N, AbuRahma AF, Cash BD, Kapoor BS, Knuttinen MG, Minocha J, et al. ACR Appropriateness Criteria. J Am Coll Radiol. 2017;14(5S):S266-S71.
